# Anticancer activity of *Momordica cochinchinensis* (red gac) aril and the impact of varietal diversity

**DOI:** 10.1186/s12906-020-03122-z

**Published:** 2020-11-25

**Authors:** Dilani Wimalasiri, Chaitali Dekiwadia, Siat Yee Fong, Terrence J. Piva, Tien Huynh

**Affiliations:** 1 School of Sciences (Biotechnology), RMIT University, PO Box 71, Bundoora, 3083 Australia; 2 Microscopy and Microanalysis Facility, school of Science Engineering and Health, RMIT University, GPO Box 2476V, Melbourne, Victoria 3001 Australia; 3 Faculty of Medicine and Health Sciences, Universiti Malaysia Sabah, Kota Kinabalu, Malaysia; 4 School of Health and Biomedical Sciences, RMIT University, PO Box 71, Bundoora, 3083 Australia

**Keywords:** Apoptosis, Breast cancer, Cytotoxicity, Melanoma, *Momordica cochinchinensis*, Necrosis, Red Gac

## Abstract

**Background:**

*Momordica cochinchinensis* (Cucurbitaceae) is a nutritionally and medicinally important fruit restricted to South East Asia with diverse morphological and genetic variations but there is limited information on its medicinal potential.

**Methods:**

*M. cochinchinensis* aril from 44 different samples in Australia, Thailand and Vietnam were extracted using different solvents and tested for its anticancer potential. Anticancer activity of *M. cochinchinensis* aril on breast cancer (MCF7 and BT474) and melanoma (MM418C1 and D24) cells were compared to control fibroblasts (NHDF). The cytotoxicity of the cells following treatment with the aril extract was determined using CCK-8 assay. Biochemical and morphological changes were analysed using flow cytometry, confocal and transmission electron microscopy to determine the mechanism of cell death.

**Results:**

The water extract from the aril of *M. cochinchinensis* elicited significantly higher cytotoxicity towards breast cancer and melanoma cells than the HAE extract. The IC_50_ concentration for the crude water extract ranged from 0.49 to 0.73 mg/mL and induced both apoptotic and necrotic cell death in a dose- and time-dependant manner with typical biochemical and morphological characteristics. The greatest cytotoxicity was observed from Northern Vietnam samples which caused 70 and 50% melanoma and breast cancer cell death, respectively.

**Conclusions:**

The water extract of *M. cochinchinensis* aril caused significant apoptosis and necrosis of breast cancer and melanoma cells, with varieties from Northern Vietnam possessing superior activity. This highlights the potential of this fruit in the development of novel anticancer agents against such tumours, with specific regions on where to collect the best variety and extraction solvent for optimum activity.

## Introduction

Cancer is a disease characterised by uncontrolled cell growth and proliferation initiated by inappropriate cell division. It is categorised as the second leading world-wide cause of death, with 14.1 million estimated cases in 2015 and is a significant health problem in both developed and developing countries [1]. Breast cancer is the second most commonly diagnosed cancer type in the world and is the most common malignancy among women [2]. However, Australia and New Zealand have the highest incidence and mortality rates from cutaneous melanoma in the world mainly due to exposure of the skin to high Ultra Violet (UV) radiation [3]. A rise in the incidence and mortality rates of these cancers has led to an increased emphasis on drug development, public health policies and awareness programs for reducing cancer [2, 4].

More than 80% of the world’s population consider traditional plant-derived medicine as their source of primary health care [5, 6]. Thus, there is a continuing need for the development of new anticancer drugs and/or drug combinations, through methodical and scientific exploration of the enormous pool of plant-based products. Currently, the most common approaches for treating cancer include chemotherapy, surgery and radiotherapy [7]. However, non-surgical therapies are associated with toxicity due to non-selective targets [8]. Plant-derived medicines have a long history of use in the treatment of cancer and over 60% of currently used anti-cancer agents originated from natural sources [5, 9, 10].

Fruits and vegetables are rich sources of phytochemicals with antioxidant, immune-modulatory and anti-cancer properties [11, 12]. Understanding the mechanism of cell death induced by phytochemicals is a key step in the development of chemo-preventive or chemo-therapeutic drugs. Phytochemicals can induce cell death by two well-known mechanisms: necrosis or apoptosis. These two forms of cell death differ from each other with respect to morphology, changes in the cell surface markers such as phosphatidylserine, altered levels of surface enzyme activity, DNA fragmentation and activation of caspases and other proteins which cleave intracellular proteins [13–16].

The fruit mesocarp and aril of *Momordica cochinchinensis* is rich in phytochemicals such as carotenoids, flavonoids and phenolics with potential pro-vitamin A, antimicrobial and anticancer activities [17]. The aril contains high levels of carotenoids such as lycopene and β-carotene [18, 19]. These carotenoids possess antioxidant, anti-inflammatory, cardio protective and anticancer effects [20–23]. Water extracts of the aril was effective against colon cancer in vivo and in vitro by inducing necrosis attributed to an unknown 35 kDa protein [24]. However, so far, it is unknown whether the aril extract is cytotoxic to other cancer cells such as breast cancer and melanoma.

*M. cochinchinensis* is genetically different and grown in diverse eco-geographical conditions [25]. This might result in variation of the phytochemical composition in the fruits and successive anticancer activity. Phytochemicals are produced as a plant’s defence mechanism, helping it adapt to both micro and macro environments, such as water stress, temperature stress, UV light and disease [26]. These metabolites can be significantly influenced by many intrinsic and external factors, such as genetic differences within species, stage of growth and development, soil fertility, availability of water and light, competition with neighbouring plants and interactions with pathogens and parasites, such as bacteria, fungi, viruses and nematodes [27]. Understanding the variability in anticancer activity of *M. cochinchinensis* aril, based on collection sites and their climatic factors, will be important for plant selection, conservation and future developments in the functional foods industry.

The aims of this study were firstly, to investigate the cytotoxicity effect of different extraction solvents on the aril of *M. cochinchinensis* fruit against melanoma (MM418C1 and D24) and breast cancer (MCF7 and BT474) cell lines. Secondly, to determine the dose- and time- dependant effect of the aril extract. Thirdly, to compare the anticancer activity of aril extracts of *M. cochinchinensis* collected from Thailand, Vietnam and Australia.

## Materials and methods

### Sample collection and carotenoid analysis

Fruits of 44 *M. cochinchinensis* samples were collected from Thailand, Southern Vietnam, Central Vietnam and Northern Vietnam growing in their natural habitats. These samples were collected with permission from private land owners, local researchers and botanists. Formal identification of plants were conducted by Dr. Sophie Parks (Department of Primary Industries Australia), Mr. Rattanapong Charntawong (Siam Golden Fruit Limited Thailand), Dr. Cuong Nguyen (Hanoi University of Agriculture Vietnam), Professor Nhut Tan Duong (Tay Nguyen Institute of Biology Vietnam) and Mr. Khoa Luu (Tay Do University Vietnam). Samples from Australia grown in green house conditions were provided by Dr. Sophie Parks from the Department of Primary Industries, New South Wales (NSW) and were used for comparison. Voucher specimens were deposited at the National Herbarium of Victoria (accession MEL2472087).

The samples were collected during their harvesting season (December–February) between 2011 and 2014. The fruits were cleaned; the aril separated from its seeds and transported to RMIT University in an insulated bag and stored at − 20 °C in darkness until required. The geographical (altitude, latitude and longitude) and ecological (rainfall of wettest and driest month, observed minimum and maximum temperature, annual temperature range) data were obtained for each province using DIVA-GIS spacial analysis software [28]. For anticancer activity analysis, 15 mature fruits were systematically selected from Thailand (*n* = 3), Southern Vietnam (*n* = 3), Central Vietnam (*n* = 3), Northern Vietnam (*n* = 3) and Australia (*n* = 3) for cytotoxicity comparisons (Table 1). The aril of the VC29 sample (Lam Dong, Central Vietnam) was selected for further testing due to its average carotenoid concentration using HPLC from a previous study [19].

**Table 1:** Eco-geographical factors and carotenoid content of *M. cochinchinensis* from Vietnam (southern, northern and central), Thailand and Australia. Bioclimatic data was obtained from DIVA-GIS spacial analysis software. Carotenoids quantified using HPLC relative to standards with HAE extracts

Country	Province	Sample code	Altitude (m)	Temperature ^o^C			Precipitation (mm)				Carotenoids mg/g	
				Max	Min	Annual range	Annual range	Annual	Wettest month	Driest month	Lycopene	β-carotene
S Vietnam	Can Tho	VS1	1	32.6	22.2	10.4	10.4	1524	254	2	0.3	1.0
	Vinh Long	VS3b	2	33.2	21.9	11.3	11.3	1413	244	2	1.3	0.8
	HCM city	VS7	6	34.7	21.1	13.6	13.6	1851	310	2	1.9	0.4
N Vietnam	Hung Yen	VN9a	71	32.2	11.6	20.6	20.6	1774	325	18	5.2	2.0
	Ha Noi	VN16	9	33.0	13.5	19.5	19.5	1700	314	14	7.5	5.3
	Hoa Binh	VN22	195	31.6	11.7	19.9	19.9	1801	352	8	5.1	5.7
C Vietnam	Lam Dong	VC28	1112	27.9	13.3	14.6	14.6	1651	248	7	6.6	1.7
	Lam Dong	VC29^a^	1112	27.9	13.3	14.6	14.6	1651	248	7	5.8	1.5
	Lam Ha	VC32	925	28.1	13.8	14.3	14.3	1658	252	8	6.3	1.8
Thailand	Nakhon Pathom	TH4	8	35.9	18.7	17.2	17.2	1237	257	6	2.3	1.5
	Samut Prakan	TH5	2	34.5	20.4	14.1	14.1	1439	325	10	2.5	0.3
	Chiang Mai	TH8	316	36.2	12.9	23.3	23.3	1163	244	7	2.9	0.2
Australia	Newcastle	A8	NA	25.0	18.0	NA	NA	NA	NA	NA	1.6	0.5
	Newcastle	A10	NA	25.0	18.0	NA	NA	NA	NA	NA	2.1	0.8
	Newcastle	A12	NA	25.0	18.0	NA	NA	NA	NA	NA	1.7	0.7

^a^: Sample was selected for use in all experiments due to the mid-range level of carotenoids

*NA* Information not available

HPLC analysis used 4 mL of the HAE extracts in HPLC injection solvent (THF:acetonitrile:methanol 50:25:25). The final solvent was filtered through 0.45 μm membrane filters and 20 μL was used for HPLC analysis. Samples were analysed using an isocratic method (90% CH_3_CN/H_2_O) on an Alltech Alltima HP C18 (250 × 4.6) 5 μm column at a flow rate of 1.0 mL/min. Analytical HPLC analyses were performed on a Dionex P680 solvent delivery system equipped with a PDA100 UV detector (operated using “Chromeleon” software). The column temperature was 30 °C and UV detection monitored at 475 nm. The concentrations of lycopene and β-carotene were quantified with reference to samples of the commercial standards of lycopene and β-carotene (Sigma Chemical, St. Louis, USA) of known concentration ranging from 10 to 400 μg/mL which were used to obtain a calibration plot. The regression equation was used to determine the lycopene and β-carotene content of the samples where, y = 1.16x - 4.369, *R*^*2*^ = 0.971 for lycopene and y = 0.540x + 0.458, *R*^*2*^ = 0.984 for β-carotene. The analyses were performed in triplicates for all the samples.

### Preparation of plant extracts

### Hexane:acetone:ethanol (HAE) extract

The frozen aril (2 g) was placed in a vessel, protected from sunlight and mixed with 100 mL of hexane:acetone:ethanol 2:1:1 extraction solvent (HAE) (Sigma-Aldrich, USA). The mixture was ultra-sonicated (Unisonics, Australia) for 30 min and 15 mL of distilled water was added to enhance phase separation, where the upper hexane layer contained the carotenoids and the bottom water layer contained hydrophilic compounds and cell debris. The dried carotenoid extract was weighed, diluted to a concentration of 11 mg/g using delivery vehicles. Since carotenoids are water insoluble, different solvents such as 0.1% DMSO (Sigma-Aldrich), 1% Ethanol, Tween 40 (20 g/100 mL in acetone) (Sigma-Aldrich) and DMEM (Dulbecco’s Modified Eagle’s Medium) (Gibco, Life Technologies, USA) media with 10% FBS (Foetal Bovine Serum) (Serana, Australia) were tested as delivery vehicles. The extract was filter sterilised using 0.45 μm filters before introduction to the cells, 10 μL of the extract was added to 96 well plates containing 100 μL of media to give a final concentration of 1 mg/mL. These HAE extracts were analysed by HPLC to determine the carotenoid content of the samples.

### Water extract

The frozen aril sample (2 g) was placed in a vessel, protected from sunlight and mixed with 100 mL of distilled water. The mixture was ultra-sonicated for 30 min, filtered through Whatman® qualitative filter paper (Grade 1) and evaporated to dryness using a rotary evaporator (Büchi Labortechnik AG, Australia). The dried crude extract was weighed, diluted to appropriate working concentrations (2.75–22 mg/mL) using milliQ water and incubated in a water-bath sonicator (Unisonics, Australia) for 10 min. The extract was filter sterilised using 0.45 μm filters and 10 μL of the extract was added to 96 well plates with 100 μL of media to give a final concentration of 0.25–2 mg/mL.

### Cell lines and culture conditions

Human breast cancer MCF7 (p53^WT^ tumour suppressor gene and caspase-3 deficient) and BT474 (mutated p53^E285K^ tumour suppressor gene) were provided by the School of Medical Sciences, RMIT University, Australia. Melanoma MM418C1 (BRAF^V600E^ oncogene) and D24 (BRAF^WT^ oncogene) cell lines were kindly supplied by Professor Nicholas Hayward, QIMR, Brisbane. The breast cancer and melanoma cells were maintained in Dulbecco’s Modified Eagle’s Medium (DMEM) and Roswell Park Memorial Institute medium-1640 media (RPMI-1640) (Gibco), respectively, supplemented with 10% FBS (Serana), 1% v/v streptomycin and penicillin (Gibco) at 37 °C in a humidified atmosphere of 5% CO_2_. Neonatal human dermal fibroblast cells (NHDF) were used as normal untransformed cells and grown in DMEM media supplemented with 10% FBS, 1% v/v streptomycin and penicillin at 37 °C in 5% CO_2_.

### In vitro cytotoxicity assay

The effect of the extracts on cell viability was determined using the Cell Counting Kit-8 (CCK-8) (Sigma-Aldrich,) according to the manufacturer’s instructions. The melanoma and breast cancer cells were seeded in 96 well flat bottom plates (Greiner Bio-One, Australia), 5 × 10^3^ cells/well and 3 × 10^3^ cells/well, respectively, along with 100 μL of fresh media. The cells were allowed to attach for 24 h before being treated with 10 μL of 11 mg/mL of the water or HAE extract to give a final concentration of 1 mg/mL. The cells were exposed to the extract either 24 or 72 h.

In order to calculate the IC_50_ concentration for the water extract, the cells were treated with 10 μL of the extract at different concentrations (2.75–22 mg/mL). After 22 h and 70 h of treatment (total time including after the CCK-8 solution was added was 24 and 72 h, respectively), 10 μL of CCK-8 solution was added to each well of the 96 well plate containing treated and control samples. The plates were incubated in 37 °C for 2 h and the absorbance was measured spectrophotometrically at 450 nm using a CLARIOstar® High Performance Monochromator Multimode Microplate Reader (BMG Labtech, Australia). The results were analysed using the BMG La Tech, MARS data analysis software (version 3.00R_2_). The data was presented as proportional viability (%) by comparing the treated cells with the untreated cells (control), using the following equation:

Cell viability (%) = (Absorbance of sample/Absorbance of control) × 100.

Two types of controls were used; the media control consisted of cultured cells in 10% FBS-containing medium alone and the vehicle control consisted of cells in 10% FBS-containing medium and 10 μL of sterile water. However, as both controls did not cause cytotoxicity, the media control was used to calculate cell viability. Only the MCF7, MM418C1 and D24 cell lines which showed the highest cytotoxic effect against the *M. cochinchinensis* aril was used for further experiments.

### Quantification of apoptosis by Annexin V labelling

The percentage of cells undergoing apoptosis was quantified using the Annexin V & Dead Cell kit (Millipore, USA). Four populations of cells can be distinguished in this assay: viable cells (Annexin V negative and 7-AAD negative), early apoptotic cells (Annexin V positive and 7-AAD negative), late-stage apoptotic and dead cells (Annexin V positive and 7-AAD positive), and cellular debris (Annexin V negative and 7-AAD positive).

The assay was performed according to the manufacturer’s instructions. Briefly, the melanoma (5 × 10^3^ cells/well) and breast cancer cells (3 × 10^3^ cells/well) were seeded in 96 well plates and permitted to adhere for 24 h at 37 °C in 5% CO_2._ The cells were treated with 1 mg/mL of the water extract for 24 and 72 h. Detached and adherent cells were collected by trypsinisation and transferred into 1.5 mL microcentrifuge tubes and centrifuged at 400 g for 5 min. The cell pellets were resuspended in 100 μL of fresh medium, to which 100 μL of Muse Annexin V & Dead Cell assay kit reagent was added. The content was mixed and incubated for 20 min at room temperature (RT) in darkness. The number of live, early apoptotic and late apoptotic/necrotic cells per 100 events were counted with the Muse Cell Analyser (Millipore). All tests and analyses were conducted in triplicate experiments.

### Detection of apoptosis and necrosis by confocal microscopy

Annexin V-FITC and propidium iodide (PI) stains (Beckman Coulter, USA) were used to detect apoptotic and necrotic cells under confocal microscopy according to the manufacturer’s instructions with slight modifications. The melanoma (5 × 10^3^ cells/well) and breast cancer cells (3 × 10^3^ cells/well) were seeded in 96 well plates and permitted to adhere for 24 h at 37 °C in 5% CO_2_. The cells were treated with 1 mg/mL of the aril water extract for 72 h. Annexin V-FITC and PI was diluted with binding buffer (Beckman Coulter) for a final concentration of 0.2 mg/mL and 0.1 mg/mL, respectively. The cells were incubated with Annexin V-FITC for 15 min and with PI for 5 min. The fluorescence images of treated and control cells of MCF7, MM418C1 and D24 cell lines were captured using a Nikon Eclipse Ti-E (inverted) confocal microscope (Nikon, Japan).

### Cell morphology analysis by phase contrast microscopy

Morphological changes to cells treated with 1 mg/mL aril water extract at 24 and 72 h time points were analysed using an inverted light microscope (Nikon Eclipse TS100). The images were captured using a Nikon digital camera (DS-Fi1) and DS-L2 control unit.

### Ultra-structural analysis of melanoma and breast cancer cells using transmission electron microscopy (TEM)

Breast cancer and melanoma cells were treated with *M. cochinchinensis* water extract (2 mg/mL) for 72 h. The treated cells were washed with 0.1 M PBS and stained with 2.5% (v/v) glutaraldehyde and 2% (v/v) paraformaldehyde in 0.1 M cacodylate buffer (pH 7.3) for 30 min. The stained cells were centrifuged (400 g for 5 min at RT), rinsed with 0.1 M sodium cacodylate buffer (pH 7.3) twice and left overnight in the same buffer. The cells were fixed with 1% (v/v) osmium tetroxide and 1.5% potassium ferrocyanide for 1.5 h, at RT and washed twice with distilled water for 10 min. Dehydration was conducted as follows; 50% (v/v) ethanol for 15 min followed by 70% (v/v) ethanol for 15 min, 90% (v/v) ethanol for 15 min, 95% (v/v) ethanol for 15 min, followed by 100% (v/v) ethanol for 30 min and repeated, finally twice with 100% (v/v) acetone for 30 min. Infiltration was carried out using a mixture of acetone and Spurr’s resin mix (1:1) on a shaker overnight at RT. The next day, new acetone: Spurr’s resin mix (1:1) was added to the cells and left for 2 h. Then, 100% Spurr’s resin was added to the cells and they were placed under vacuum for 2 h. This was followed by exchanging to fresh resin and continuing the infiltration for 2 h. Finally, the cells were embedded and cured at 70 °C for 24 h. Sectioning was done using an UCT ultra-microtome (Leica Ultracut, Germany) to produce < 1 μm thin sections. The thin sections were washed with distilled water and dried on blotting paper. The sections were examined at 80 kV with a JEOL1010 (Japan) transmission electron microscope and images were obtained using Gatan Orius SC600 CCD Camera equipped with Gatan Microscopy Suite software version 2.3.

### Impact of varietal differences of *M. cochinchinensis* on cytotoxicity

The crude water extracts from 15 samples, consisting of three samples each region from Thailand, Vietnam (Northern, Central and Southern) and Australia were tested for their cytotoxicity using the CCK-8 assay. Breast cancer (MCF7) and melanoma (MM418C1 and D24) cells were plated in 96 well plates at a cell density of 3 × 10^3^ cells/well and 5 × 10^3^ cells/well, respectively, and were treated with 1 mg/mL water extract. Neonatal human dermal fibroblast cells (NHDF) were not tested since the previous experiments showed that the aril water extracts did not elicit any cytotoxic effects on these cells. Control cells were cultured in 10% FBS- containing tissue culture medium only. The plates were incubated for 72 h after adding the extract and the readings were taken as described previously. All tests and analyses were done for three individual experiments performed in duplicate.

### Statistical analysis

The results were analysed with the one-way analysis of variance (ANOVA) using Minitab statistical software (version 17). For normally distributed data, the means were compared using the one-way analysis of variance (ANOVA) and Fisher’s post-hoc test. Pearson’s correlation was used to test the correlation between cytotoxicity and eco-geographical parameters. Statistical values of *P* < 0.05 were considered as significantly different. The inhibitory concentration (IC_50_) for cytotoxicity was derived from a nonlinear regression model (curve fit) based on a sigmoidal dose response curve (variable) and computed using GraphPad Prism version 6. Three component principal component analysis (PCA) for anticancer activity of the aril water extract of *M. cochinchinensis* was carried out using Minitab statistical software (version 17).

## Results

### Effect of extraction solvent (HAE vs water) of *M. cochinchinensis* on breast cancer and melanoma cell viability

**Fig. 1:**
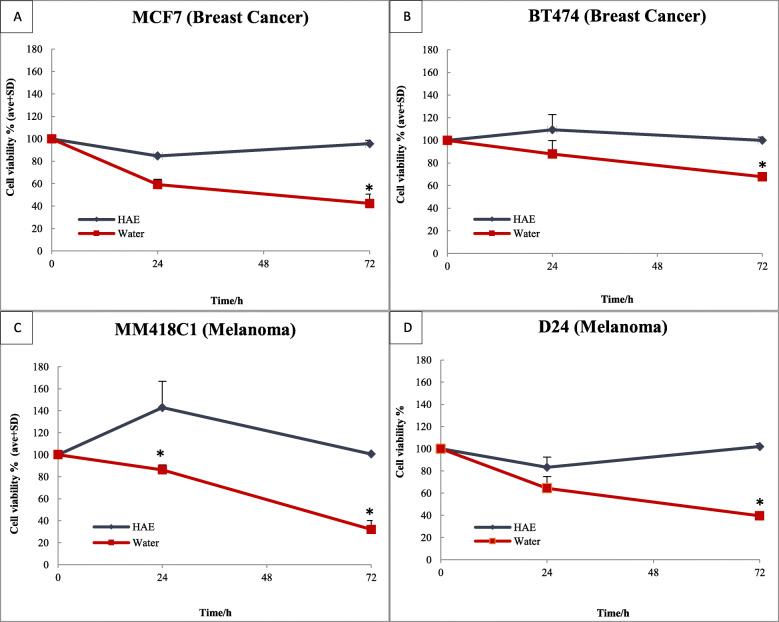
Effect of *M. cochinchinensis* aril extracted with HAE or water on the viability of MCF7 (**a**), BT474 (**b**) breast cancer cells, and MM418C1 (**c**), D24 (**d**) melanoma cells after 24 and 72 h. 0 h indicated the time the extracts were added. Results were representative of three independent experiments. The values shown were average + SD compared to controls. * indicated significance at *p* ≤ 0.05 between HAE or water extracts at the same time point

Carotenoids are water insoluble. Therefore, different vehicle controls such as 0.1% DMSO, 1% Ethanol, Tween 40 and DMEM (Dulbecco’s Modified Eagle’s Medium) media with 10% FBS (Foetal Bovine Serum) were tested but only DMEM media supplemented with 10% FBS was effective in dissolving the carotenoids in the aril extract of sample VC29* which was from the Lam Dong region of Central Vietnam. The amount of lycopene and β-carotene in the HAE extract was 5.8 mg/g and 1.5 mg/g aril, respectively (Table 1). When the breast cancer and melanoma cells were exposed to HAE extracts for 24 h, this reduced the cell viability of the MCF7 breast cancer and D24 melanoma by 15% but had no effect on BT474 breast cancer and MM418C1 melanoma (Fig. 1). Similarly, prolonged exposure of the HAE extract (72 h) showed cytotoxicity similar to that seen at 0 and 24 h, suggesting that carotenoids in *M. cochinchinensis* had low activity.

**Fig. 2:**
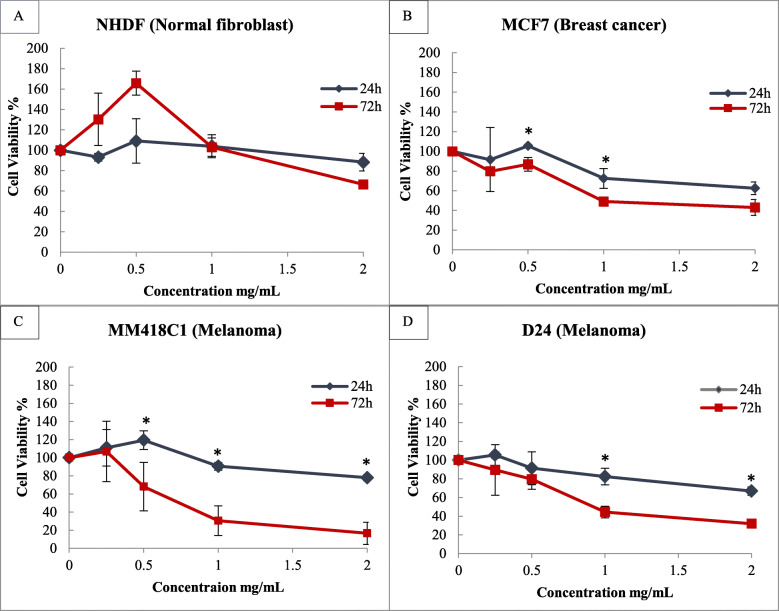
Effect of exposure time and concentration of *M. cochinchinensis* aril water extract on cell viability. Aril water extracts (0–2 mg/mL) were added to normal human dermal fibroblast (NHDF) (**a**), MCF7 (**b**) breast cancer cells, and MM418C1 (**c**), D24 (**d**) melanoma cells and measured at 24 and 72 h. Results were the means ± SD of three independent experiments done in triplicate (*n* = 9). * indicated significance at *p* ≤ 0.05 between the two time points

The water extract induced a time-dependant cytotoxicity in the breast cancer and melanoma cell lines with the highest level of activity seen at 72 h (Fig. 2) and cells underwent morphological changes. This extract caused a 15–40% loss in cell viability in both breast cancer and melanoma cells at 24 h but no visible morphological changes were observed (Fig. 2). At 72 h, the water extracts reduced cell viability by up to 70% in the MM418C1 cells. Treatment of the cells resulted in distinct morphological changes (detachment from the substrate, rounding of cells) in both MM418C1 and MCF7 cells. This reduction of cell viability was more than twice that seen at 24 h which suggest that the extract elicited a time-dependant activity in both melanoma cell lines (MM418C1 and D24) and the MCF7 breast cancer cell lines. However, the breast cancer cell line BT474, was less sensitive to treatment with only 30% cell death observed at 72 h (Fig. 2), which was lower than seen for the other cell lines. Therefore, both melanoma cell lines (MM418C1 and D24) and only one breast cancer cell line (MCF7) was used for further experiments. Human dermal fibroblasts (NHDF) represented a non-neoplastic cell line as the control and remained above 66% even with the high extract dose of 2 mg/mL at 72 h.

The water extract elicited a dose-dependent cytotoxicity on the melanoma (MM418C1 and D24) and breast cancer (MCF7) cells. The MM418C1 cells were the most sensitive with an 80% loss in cell viability seen at 72 h for cells treated with 2 mg/mL water extract (Fig. 2). At 24 h, low concentrations (0.25 and 0.5 mg/mL) of the water extract displayed a hormetic effect on all the cell lines tested. Higher concentrations (1–2 mg/mL) of the water extracts caused cytotoxicity with up to a 30% loss in cell viability (Fig. 2) observed at 24 h, which was less than that seen at 72 h. Therefore the 72 h time point was selected to determine the IC_50_ value because it showed the highest reduction in cell viability for all the examined cell lines (Fig. 2). MM148C1 melanoma cells were the most sensitive cell line having an IC_50_ of 0.49 mg/mL. The IC_50_ for the MCF7 breast cancer cells was 0.59 mg/mL and 0.73 mg/mL for the D24 melanoma cells. The IC_50_ value for the human dermal fibroblast cells was > 2 mg/mL (Fig. 2) and at low concentrations (< 1 mg/mL) the extract was shown to exert a hormetic effect where cell growth benefitted more than at higher doses. The results suggest that the *M. cochinchinensis* aril water extract possessed a selective cytotoxicity towards neoplastic cells but was not toxic to fibroblasts, except at high concentrations.

### Quantification of the mechanism of cell death

**Fig. 3:**
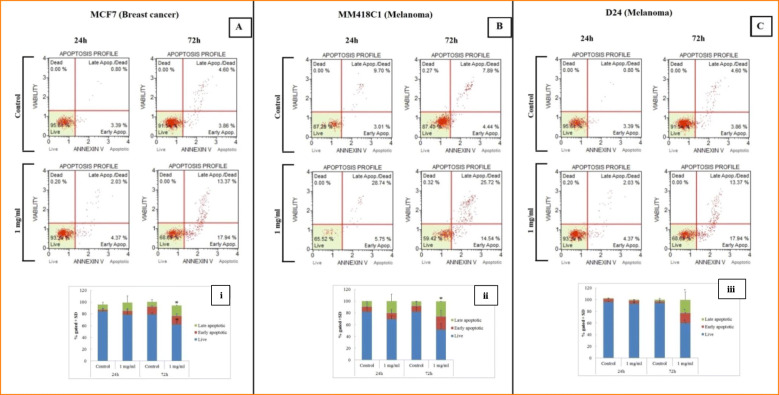
Time dependant apoptotic effect of *M. cochinchinensis* aril water extract (1 mg/mL) on MCF7 breast cancer (**a**), MM418C1 (**b**) and D24 melanoma cells (**c**) treated for 24 and 72 h. Following exposure, the cells were treated with AnnexinV/ 7AAD and analysed by muse flow cytometry. Percentages of live and apoptotic cells (early and late) were shown in histograms (i-iii) which were representative of three independent experiments (*n* = 3). * indicated significance at *p* ≤ 0.05 compared with untreated controls

The *M. cochinchinensis* water extract induced apoptosis in the breast cancer (MCF7) and melanoma (MM418C1, D24) cells in a time-dependant manner as detected by muse flow cytometry after the treatment with 1 mg/mL water extract for 72 h. Annexin V was bound to the externalised plasma membrane of the cells, as shown by the right shift of the scatter plot (Fig. 3) compared to that of non-treated cells (control cells) indicating early apoptosis. This was confirmed by the Annexin V-FITC/ PI double staining method using confocal microscopy, which showed that a greater number of cells were stained green, indicating apoptosis (Fig. 3). The percentage of viable cells decreased significantly in a time-dependent manner when all three cell lines were treated with the water extract (Fig. 3). The highest level of cell death (apoptosis and necrosis) was observed for the MM418C1 cells treated for 72 h (47.9%) with the water extract and this was in agreement with that seen in the cytotoxicity assay (Fig. 2).

**Fig. 4:**
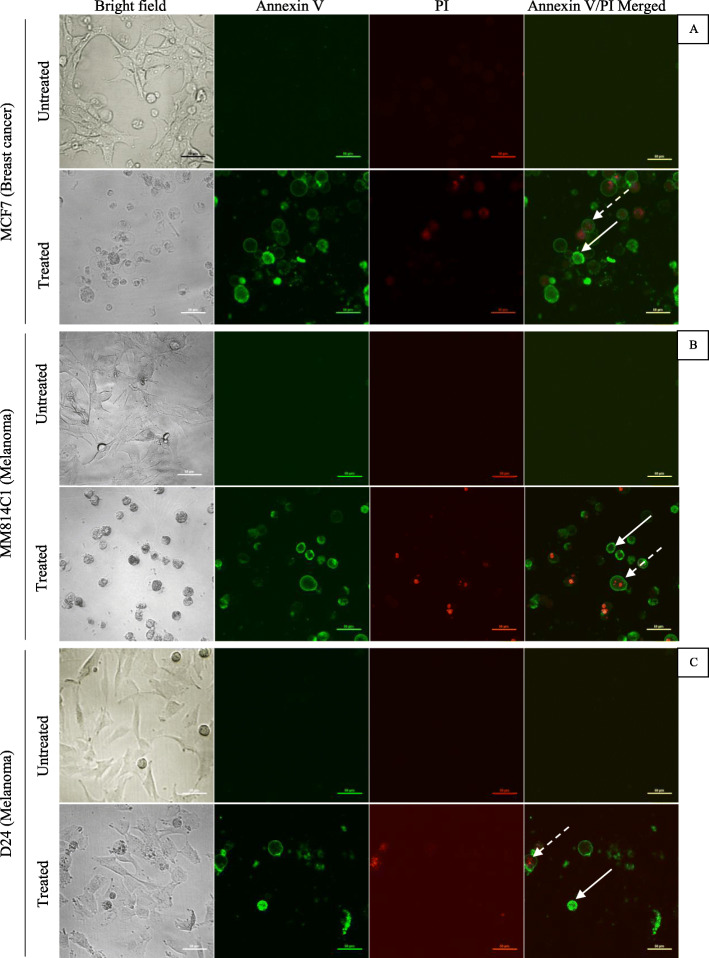
Effect *M. cochinchinensis* aril water extract (1 mg/mL) on MCF7 breast cancer (**a**), MM418C1 (**b**) and D24 (**c**) melanoma cells untreated (control) and treated (extract) at 72 h, as detected by the Annexin V-FITC (green) /PI (red) double staining assay observed under confocal microscopy. Solid-line arrows indicated early apoptotic cells and dotted-line arrows indicated late apoptotic/necrotic cells

The percentages of late apoptotic/necrotic (Annexin V positive and 7-AAD positive) cells in the breast cancer and melanoma cell lines were significantly different to that of their untreated controls (Fig. 4). This was also observed by confocal microscope analysis where the cells were stained with green (Annexin V) and red (PI) stains (Fig. 4). However, early apoptotic events (Annexin V positive and 7-AAD negative) were not significantly different for the MCF7 and MM418C1 cell lines than for the corresponding controls (Fig. 4). The water extract-treated D24 melanoma cell line had significantly higher early apoptotic events at the 72 h time point, than the corresponding untreated control (Fig. 4). This was also confirmed with the Annexin/PI staining method where the number of green (Annexin V) stained cells was higher than for those stained red (PI) which indicated apoptosis and necrosis, respectively (Fig. 4).

### Ultrastructure analysis of *M. cochinchinensis* water extract treated breast cancer (MCF7) and melanoma (MM418C1 and D24) cells

**Fig. 5:**
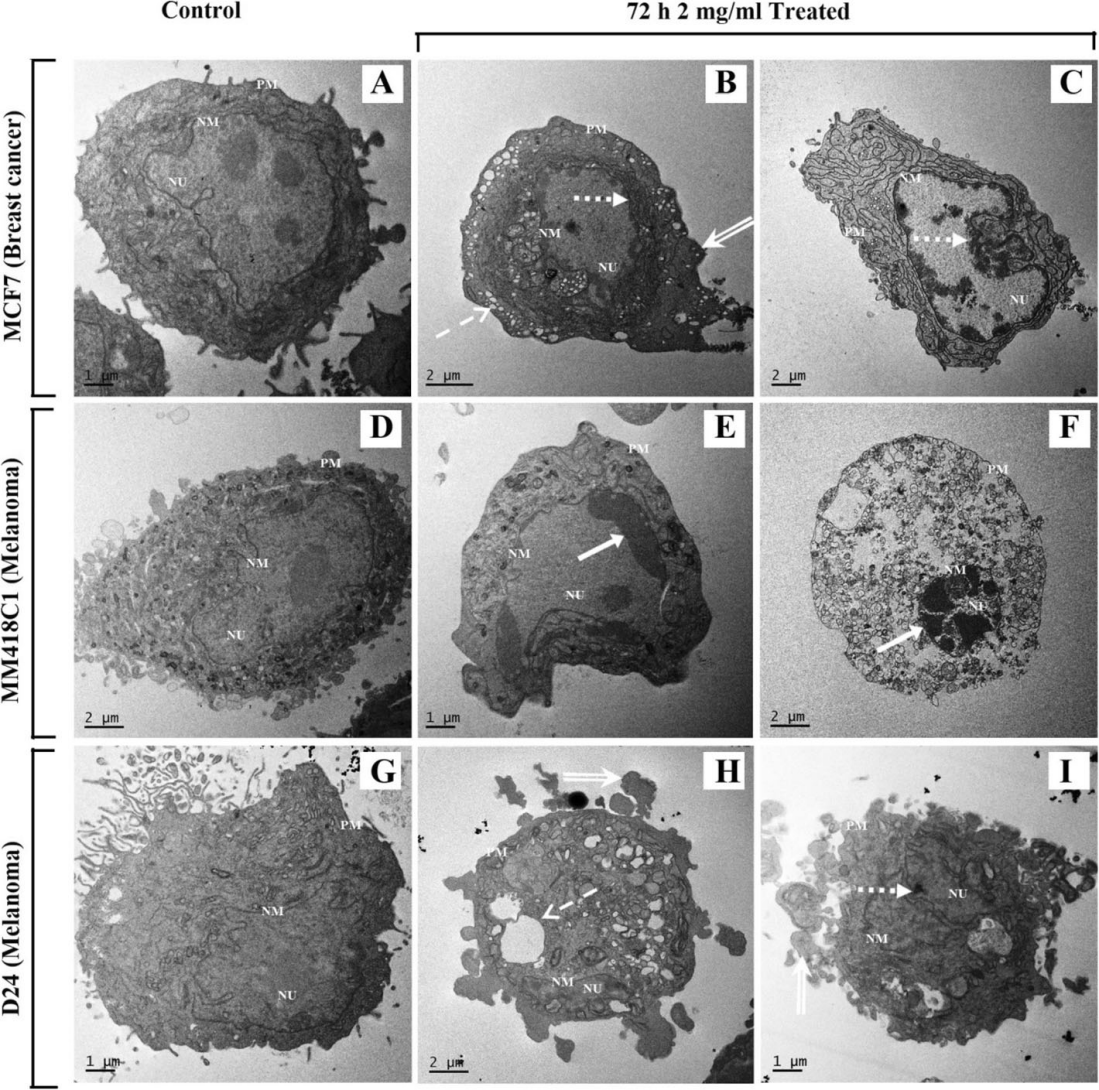
Ultrastructure analysis of breast cancer and melanoma cell lines treated with *M. cochinchinensis* aril water extract (2 mg/mL). **a-c:** Control (**a**) and 2 mg/mL treated MCF7 cells with morphological alterations at 72 h. **d-i:** Control (**d**, **g**) and 2 mg/ml treated MM418C1 (**e**-**f**) and D24 (**h**-**i**) melanoma cells with apoptotic and necrotic cell death. Solid-line arrow: Chromatin condensation. Dotted-line arrow: Margination of chromatin. Hatched-line arrow: Vacuolisation. Double-line arrow: Cell membrane blebbing. NU: Nucleus, NM: Nuclear membrane, PM: Plasma membrane

The effects of 2 mg/mL *M. cochinchinensis* water extract on the morphology of the cells treated for 72 h were observed under electron microscopy (Fig. 5). The water extract induced clear morphological changes in both breast cancer and melanoma cell lines. The untreated MCF7 breast cancer cells possessed a large nucleus with distinct nucleoli and did not have any peripheral heterochromatin (Fig. 5a). After treatment with the water extract for 72 h, these cells showed loss of microvilli, reduction of cytoplasmic volume and formation of cytoplasmic vacuoles as well as blebbing of plasma membranes (Fig. 5b-c). Furthermore, chromatin condensation was observed on the nuclear membrane (Fig. 5b-c) which displayed the characteristics of early apoptotic cells, however there were also vacuolisation of the cytoplasm (Fig. 5b) as well as a swollen endoplasmic reticulum, which was indicative of necrosis. This suggested that the water extract of *M. cochinchinensis* contained compounds that can induce apoptosis and necrosis in MCF7 breast cancer cells.

The untreated MM418C1 cells possessed large nucleoli within the nucleus with no peripheral heterochromatin (Fig. 5d). After 72 h treatment with the water extract, apoptosis was observed indicated by the chromatin condensation, shrinkage of the cytoplasmic volume and blebbing of the cytoplasm (Fig. 5e). Furthermore, the late stage of the apoptotic or secondary necrotic types of cell death was also observed, as seen by the presence of highly condensed nuclei and ruptured cellular organelles in some cells (Fig. 5f).

However, in the D24 melanoma cell line, clear chromatin condensation in the nucleus was not observed but the treated cells were different from the untreated control, as seen by the loss of microvilli on the plasma membrane, blebbing and cell shrinkage (Fig. 5g-i) [29]. The nuclear chromatin was also marginated and less electron dense with highly convoluted nuclei, which was observed in the early stages of apoptosis [29]. Furthermore, these morphological changes agreed with the results of the Annexin V/7-AAD assay, which showed a high percentage of early apoptosis in D24 cells (Fig. 4), following treatment with the water extract.

### Impact of varietal differences of *M. cochinchinensis* on cytotoxicity

The effect of 1 mg/mL water extracts from *M. cochinchinensis* collected from different regions were tested on the MM418C1, D24 and MCF7 cancer cell lines. The cytotoxicity of these extracts was variable and significantly affected by varietal differences ranging from 0 to 71% loss in cancer cell viability (Table 2). The water extracts obtained from Northern and Central Vietnam significantly reduced cell viability and subsequently cell growth by up to 70% relative to untreated controls. However, not all samples from Central and Northern Vietnam displayed similar high levels of activity and this could be due to the genetic differences between those samples. The least active samples were from Southern Vietnam on MCF7, MM418C1 and D24 cells (Table 2). The extracts from this region had little or no cytotoxic effect, and in some cases, they elicited a hormetic effect. The anticancer activity of these water extracts on these cell lines was not correlated (*P* > 0.05) to their lycopene and β-carotene concentrations, which suggested that these were not the active compounds present in the extracts.

**Table 2:** Comparison of cancer cell viability treated with 1 mg/mL aril water extract after 24 h from Vietnam (Southern, Central and Northern), Thailand and Australia using CCK-8 assay. Fisher’s LSD values were generated from un-transformed cell viability data of MM418C1 (df = 14, *F* = 10.42, *p* = 0.000), D24 (df = 14, *F* = 15.58, *p* = 0.000) and MCF7 (df = 14, *F* = 2.22, *p* = 0.069) cell lines. The results were averages of three individual experiments which were performed in duplicate

Fruit origin		Viability of cancer cells (Average % + SE)		
Country	Region	MM148C1	D24	MCF7
Vietnam (VS)	Can Tho	127.5 + 10.4^*^	102.4 + 3.5^††^	98.1 + 17.4
	Vinh Long	130.6 + 8.1^*^	99.5 + 5.3^††^	102.6 + 12.1
	HCM City	123.5 + 14.3^*^	97.4 + 6.5	100.4 + 15.1
Vietnam (VN)	Hung Yen	35.7 + 5.5^*^	52.7 + 3.3	74.5 + 1.1
	Ha Noi	28.9 + 2.4^*^	39.9 + 1.1^††^	49.9 + 0.8^†^
	Hoa Binh	113.3 + 14.2^*^	71.7 + 0.9	101.3 + 4.8
Vietnam (VC)	Lam Dong	30.9 + 3.3^*^	45.5 + 1.7^††^	62.3 + 4.3
	Lam Dong	44.2 + 8.6	57.0 + 3.9^††^	67.7 + 6.5
	Lam Ha	109.9 + 13.9^*^	85.3 + 9.8^††^	116.6 + 11.4^†^
Thailand (TH)	Nakhon Pathom	106.1 + 7.0	102.4 + 3.4	71.2 + 4.5
	Samut Prakan	84.1 + 11.1	51.8 + 3.4	75.3 + 3.8
	Chiang Mai	76.9 + 4.1^*^	50.3 + 2.0	78.7 + 4.3
Australia (AU)	Newcastle	62.1 + 5.7^*^	39.8 + 5.1^††^	84.5 + 1.9
	Newcastle	79.3 + 3.8^*^	49.2 + 0.04	74.4 + 5.4
	Newcastle	77.1 + 0.8	48.0 + 0.9	75.7 + 1.4

*VS* Southern Vietnam, *VN* Northern Vietnam, *VC* Central Vietnam

*, †† and †: statistical significance at *p* ≤ 0.05 on cell viability of MM418C, D24 and MCF7, respectively based on fruit collection sites

**Fig. 6:**
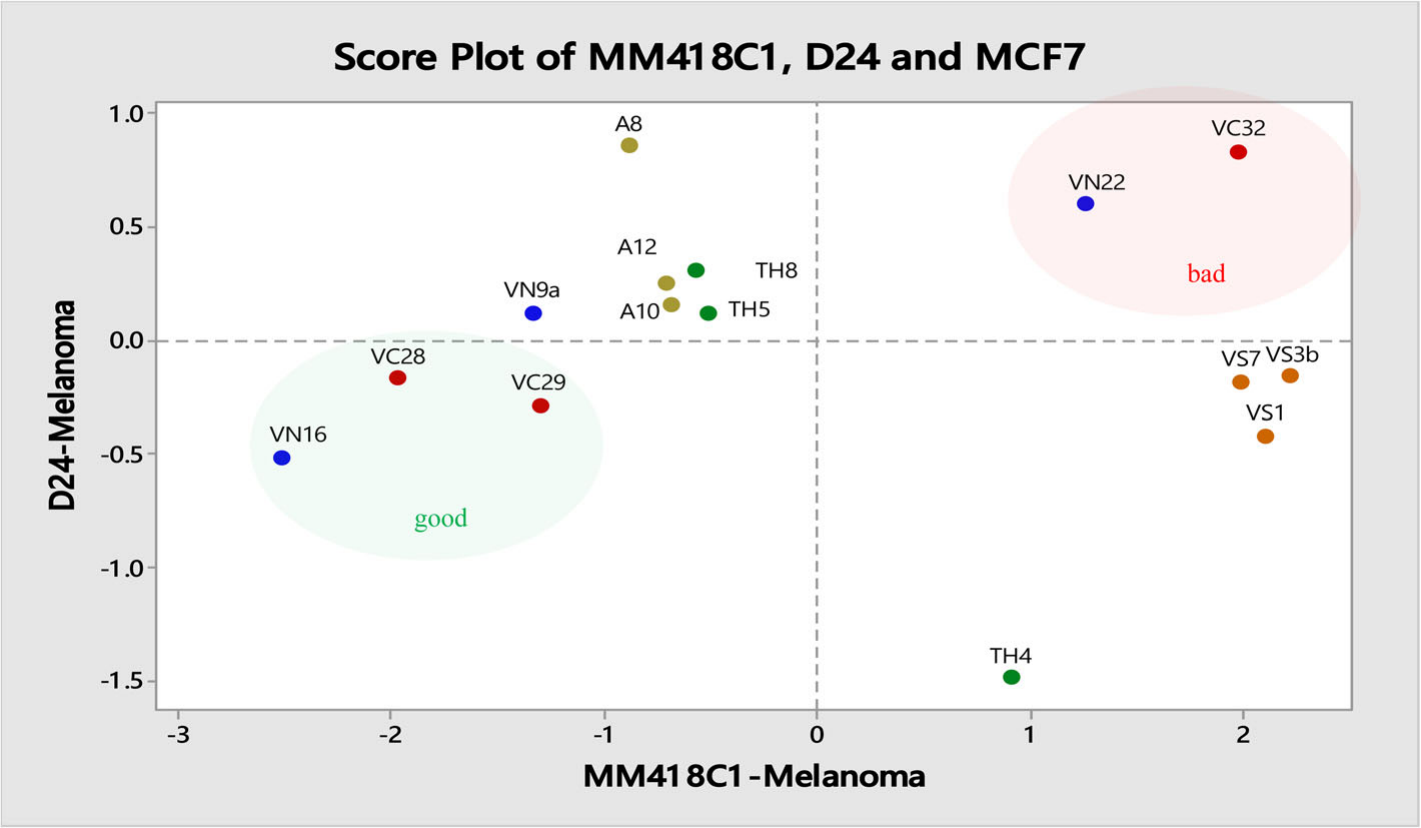
Score plot of the anticancer activity of 15 *M. cochinchinensis* aril water extract against D24 and MM418C1 cells constructed using the Principal component analysis method (PCA) with Minitab statistical software (Version 17). Country of collection was shown by different colours; orange = Southern Vietnam, blue = Northern Vietnam, red = Central Vietnam, green = Thailand, olive green = Australia

Anticancer activity of the 15 *M. cochinchinensis* water extracts on melanoma (MM418C1 and D24) and breast cancer (MCF7) cells was analysed using three component PCA analysis and presented on a 2-D graph (MM418C1 versus D24 anticancer activity) (Fig. 6). The samples with the highest anticancer activity against all three cell lines were from Northern and Central Vietnam (VN16, VC28 and VC29) and they were clustered in the bottom left quadrat of the PCA grouping equivalent to low cell viability on all cell lines. The Southern Vietnam samples clustered on the bottom right quadrat equivalent to D24 cell-sensitivity but MM418C1 cell-resistance (Fig. 6). The samples from Australia and Thailand clustered in the top left quadrat equivalent to D24 cell-resistance and MM418C1 cell-susceptibility (Fig. 6). This clustering based on the anticancer activity of *M. cochinchinensis* aril water extracts was random and did not group the samples based on geographical origin or varietal differences as did for morphology and genetic analyses [19, 25].

The samples from Australia were excluded from the analysis of correlation between carotenoid content and eco-geographical factors since they were grown in controlled environments compared to those samples obtained from Vietnam and Thailand which were exposed to environmental variations in their natural habitats (Table 3). The water extracts from the plants grown in cooler temperatures had higher cytotoxicity against MM418C1 (*r* = 0.67, *P* = 0.017) and D24 cell lines (*r* = − 0.69, *P* = 0.013) than for plants grown in warmer climates, as seen by the high correlations between the minimum temperature and cell viability (Table 3). Furthermore, the samples collected from regions with high precipitation in the driest month correlated with high cytotoxicity on MM418C1 (*r* = − 0.73, *P* = 0.007) and D24 (*r* = − 0.72, *P* = 0.009) cancer cells (Table 3). This can be interpreted as > 48% of variability in cytotoxicity of MM418C1 and D24 was due to minimum temperature and high precipitation in the driest month (Table 3). However, aril collected from different eco-geographical conditions did not show any significant correlation in their cytotoxic effect of MCF7 cells (Table 3).

**Table 3:** Pearson correlation coefficients (*r*) and coefficient of determination (*r*^*2*^) between eco-geographical factors (maximum and minimum temperature, annual temperature range, elevation and precipitation) and cell viability of *M. cochinchinensis* aril water extract on breast cancer (MCF7) and melanoma (MM418C1 and D24) cells

Factor	Details	Correlation (*r*) with cell viability %			Coefficient of determination (*r*^*2*^)		
		MCF7	MM418C1	D24	MCF7	MM418C1	D24
Temperature	Minimum	0.33	0.67^*^	0.69^*^	–	0.45	0.48
	Maximum	−0.04	−0.34	−0.23	–	–	–
	Range	−0.39	0.46	−0.57	–	–	–
Elevation	Elevation	0.08	−0.40	−0.32	–	–	–
Precipitation	Annual	0.13	−0.19	−0.13	–	–	–
	Driest month	0.51	−0.73^*^	−0.72^*^	–	0.53	0.51
	Wettest month	0.05	−0.09	−0.26	–	–	–

^*^Significant at *P* < 0.05

## Discussion

### Cytotoxicity effect of water versus HAE extracts

The water extract from *M. cochinchinensis* aril elicited significantly higher cytotoxicity towards breast cancer (MCF7) and melanoma (MM418C1 and D24) cell lines than did the corresponding HAE extract. The activity of the water extracts might be due to the presence of active proteins as mentioned in a previous study [24]. The cytotoxicity of the water extract on these cell lines was low, with IC_50_ ranging between 0.49–0.73 mg/mL. The level of activity in the extract used in this study is more cytotoxic than seen in an earlier study where an IC_50_ value of 1.25 mg/mL for colon 26–20 adenocarcinoma cells was determined [24]. The discrepancy between these values may be due to several reasons, such as the differences in the plant material (collection site, variety), the post-harvest methods (storage, extraction, processing, and detection) or variation in cell lines and should be the focus of future research to determine which factor(s) specifically enhance anticancer activity. At low concentrations, the extract enhanced cell proliferation which could be due to the presence of different groups of natural compounds possessing a wide range of activities including hormetic responses.

The water extract displayed a time- and dose-dependent cytotoxicity, where the MM418C1 melanoma cell line was the most sensitive, having a IC_50_ value of 0.49 mg/mL at 72 h. This cell line possessed an oncogene mutation in the BRAF gene (BRAF^V600E^), which coded for the protein B-Raf (a protein kinase of the mitogen-activated protein kinase (MAPK) pathway) that normally regulates cell growth, proliferation and differentiation [30, 31]. This mutation is common in a large proportion (40–60%) of melanomas [30, 31]. Furthermore, the BRAF mutated MM418C1 cell line was more sensitive to the water extract than the D24 cells, which possessed the wild type BRAF gene. Thus, this suggested that the water extract might contain compounds that inhibited BRAF^V600E^, acted on the RAS-mediated signalling pathway (MAPK/ERK) or increase specific cell cycle stages leading to apoptosis [32]. Similarly, the water extract was cytotoxic to the MCF7 cell line with an IC_50_ value of 0.59 mg/mL at 72 h. This cell line is ER-positive (oestrogen receptor- positive) and ~ 70% of human breast cancers, which are hormone-dependent and ER-positive [32]. Thus, this study indicates the necessity to identify the active ingredients in the water extract of *M. cochinchinensis* aril, which could be developed as promising therapeutical agents for melanoma and breast cancer treatments.

Carotenoids are strong antioxidants and have anticancer activity as crude extracts or in purified form on prostrate, colon and breast cancer [33, 34]. The aril of *M. cochinchinensis* has the highest content of lycopene and β-carotene among known fruits and vegetables [35]. The highest detected levels of these carotenoids in HAE extracted samples were from fruit grown in Northern Vietnam (Table 1). Carotenoids are fat soluble and commonly extracted using organic solvents. The HAE extraction solvent used in this study contained high carotenoids but this was less effective on both breast cancer and melanoma cell lines than the water extract. This was in agreement with that observed in a previous study [24]. However, the low activity might also be due to the poor solubility of carotenoids in aqueous media, especially because of the high level of oil in the aril of *M. cochinchinensis* [35, 36]. This suggested the presence of other beneficial compounds such as proteins or lipids [17] that stimulate growth at low doses, which toxicologically is defined as a hormetic effect. The effective concentrations indicated in this study are realistic and comparable to the listed doses of other plant extracts where concentrations of such elicited cytotoxic effects varied from 8 μg/mL to 15 mg/mL. In many natural products bioprospecting studies, a dose of 1 mg/mL was used but it is highly likely that in vivo, this dose would be less effective.

### Water extract caused apoptosis and necrotic cell death in breast cancer and melanoma cells

The water extract from *M. cochinchinensis* aril induced cell death in melanoma and breast cancer cell lines at 72 h, as detected by the CCK8 assay, flow cytometry and confocal microscopy. The two well-known cell death mechanisms are apoptosis and necrosis, which possess distinct morphological and biochemical differences. During the initiation of cell death, cells lose contact with their neighbouring cells and adherent cells detached from the surface and became round. Further in the process, the structural integrity of the plasma membrane was lost, the final endpoint at which a cell can no longer maintain its discrete identity from the environment [37]. These cells can be measured biochemically as the release of cytosolic enzymes, including lactate dehydrogenase, and the uptake of membrane-impermeant dyes, such as propidium iodide and 7-aminoactinomycin D (7-AAD) [38]. In this study, cell death was measured using the CCK-8 assay and the Annexin V/7-AAD flow cytometry methods but these methods did not confirm the mechanism by which these cells died. It is unknown whether the cells traverse through the cell cycle or accumulate at the G1/S and/or G2/M checkpoints, and as such further investigation of the effect of the extracts on the cell cycle dynamics of the treated cells is warranted.

Morphological hallmarks of apoptosis in the nucleus are chromatin condensation and nuclear fragmentation, which can be observed under transmission electron microscopy [39]. Several stages of nuclear apoptosis can be distinguished: in the first stage, there are rippled nuclear contours and partial chromatin condensation; the second stage is distinct in having marked peripheral chromatin condensation; and in the third stage, formation of nuclear small bodies takes place [29].

The water extract induced complex responses with different nuclear morphological features, which were dependant on the cell lines analysed. The melanoma cell line, MM148C1, displayed clear apoptotic morphological features with peripheral chromatin condensation, while the D24 melanoma and MCF7 breast cancer cell lines only displayed partial chromatin condensation. The lack of apoptotic nuclei in the MCF7 cell line was expected since this cell line was caspase-3 deficient [40]. Apoptosis is typically accompanied by the activation of a class of death proteases called caspases. Among these caspases, caspase-3 is essential for the nuclear morphological changes (e.g. chromatin condensation) that occur during apoptosis [41, 42].

Crude extracts can induce complex responses in cancer cells due to the presence of more than one bio-active molecule, each of which can trigger different cell death mechanisms. It is also possible for a certain dose of death-inducing agents, to induce both apoptosis and necrosis simultaneously in cells [43, 44]. In this study, although early apoptotic nuclei were observed in cells, necrotic cell death was also likely and indicated by the cytoplasmic vacuolisation (Fig. 5b and h), rupture of cellular organelles (Fig. 5f) and swelling of the endoplasmic reticulum (Fig. 5c). This suggested a combination of death modes and the presence of different classes of compounds in the extract, and further fractionation of the extract is needed to characterise the active compounds and specify its action.

### Anticancer activity was influenced by eco-geographical differences of *M. cochinchinensis*

Geographical distribution appeared to influence the anticancer activity of *M. cochinchinensis* extracts. Cytotoxicity was significantly different based on the collection site of the fruit, with the most active sample from Ha Noi province in Northern Vietnam. The anticancer activity determined by cytotoxicity varied widely, causing up to 70% cell death. All the samples analysed were genetically different based on molecular markers and therefore, the observed variation in cytotoxicity might be due to genetic diversity. Genetic variation within a species can give rise to different compositions of biologically active secondary metabolites as reported in other medicinal plants [45, 46] but was unknown for *M. cochinchinensis*.

Fruits originating from the same plant with an identical phytochemical composition will most likely possess the same level of cytotoxicity. The three samples from Australia originated from a single fruit and therefore had low genetic variation [25], which could explain why these samples displayed similar levels of cytotoxicity. This suggested that genetics might be a predominant factor and that maintaining the same genetics could assure the consistency of bioactivity of the best genotypes. Interestingly however, although the Australian *M. cochinchinensis* variety originated from Southern Vietnam [25], it was grown in temperature-controlled glasshouses at 25 °C, which may explain why the Australian samples showed a higher cytotoxicity against the melanoma cell lines than samples from where the variety originated suggesting that variables other than genetics are responsible and the bioactivity could be improved by optimised cultivation strategies. The clustering based on anticancer activity of *M. cochinchinensis* aril water extract did not group the samples based on geographical origin or varietal differences as did for morphology and genetic analyses [25]. This might be due to other factors which were responsible for variations in anticancer compounds such as soil nutrition, micro-climatic differences and environmental differences. Furthermore, the lack of differentiation of clustering based on geographical origin from the PCA analysis might be due to the low number of samples from each location analysed in this study.

The samples collected from cool climates with a high precipitation rate during the driest month correlated with a high cytotoxicity of the aril extract towards the melanoma cells but not the breast cancer cells. This was expected since environmental stress conditions such as temperature and drought [47, 48] and processing protocols [46, 49] can result in production and detection of higher amounts of secondary metabolites in other plant species. However, more experiments are necessary (e.g. the same genotypes grown in different climates/environments) to confirm the relative dominance of eco-geographic and genetic influences on the anti-cancer activity of *M. cochinchinensis*. Future research focusing on the micro-climate of the plant growth sites is necessary to confirm the environmental influence on the anticancer activity of *M. cochinchinensis*.

## Conclusion

Water extraction of the aril from *M. cochinchinensis* was cytotoxic to breast cancer and melanoma cells. The water extract had significantly higher (> 70% cell death) cytotoxicity than the HAE extract, indicating that the active compounds were water soluble and not carotenoids, which were water insoluble; and this was in agreement with a previous study [24]. The water extract elicited both apoptotic and necrotic cell death mechanisms indicating that the extract contained more than one type of bio-active molecule, as was expected from a crude extract. Further studies on apoptosis (p53, Bcl-2, caspase-3) or necrosis (cyclin A, CDK2) as well as cell cycle related genes and proteins will provide more information on the cellular death pathways involved in the cytotoxicity of the aril water extract, which will improve the understanding of the mechanism of action of the anticancer compounds.

The fruit of *M. cochinchinensis* has potential to prevent cancer due to its high carotenoid content as well as other possible anticancer compounds that have not yet been fully characterised. The results of this project suggest that the Lam Dong province of Central Vietnam as well as the Ha Noi province of Northern Vietnam possess genotypes with high anticancer activity. This should be considered for the possibility of introducing *M. cochinchinensis* to the market as a chemo-preventive fruit in the future. For this commercial development, plants from these regions should be selected for future breeding programs. Furthermore, the anticancer activity was influenced by temperature and precipitation, where samples collected from locations with < 14 °C minimum temperatures and high precipitation rates during the driest month had high anticancer activity. This indicated that these environmental conditions could be manipulated to obtain higher bioactive compounds, which might contribute to optimised anticancer activity.

## Data Availability

All data and materials are contained and described within the manuscript.

## Abbreviations

7-AAD: 7-Aminoactinomycin D
ANOVA: Analysis of variance
CCK-8: Cell counting kit-8
DMEM: Dulbecco’s modified eagle medium
DMSO: Dimethyl sulfoxide
IC_50_: Half maximal inhibitor concentration
FBS: Foetal bovine serum
FITC: Fluorescein isothiocyanate
MeOH: Methanol
PCA: Principal component analysis
PI: Propidium iodide
RPMI: Roswell park memorial institute medium
SE: Standard error
SD: Standard deviation
TEM: Transmission electron microscopy
